# Comparative Pharmacokinetics of Gallic Acid, Protocatechuic Acid, and Quercitrin in Normal and Pyelonephritis Rats after Oral Administration of a *Polygonum capitatum* Extract

**DOI:** 10.3390/molecules24213873

**Published:** 2019-10-27

**Authors:** Yong Huang, Zuying Zhou, Wu Yang, Zipeng Gong, Yueting Li, Siying Chen, Yonglin Wang, Aimin Wang, Yanyu Lan, Ting Liu, Lin Zheng

**Affiliations:** 1 State Key Laboratory of Functions and Applications of Medicinal Plants, Guizhou Provincial Key Laboratory of Pharmaceutics, Guizhou Medical University, 4 Beijing Road, Guiyang 550004, China; mailofhy@126.com (Y.H.); zu_ing@163.com (Z.Z.); hhyangwu@163.com (W.Y.); gzp4012607@126.com (Z.G.); nhwslyt@163.com (Y.L.); Siying.chen@kcl.uk.ac (S.C.); gywyl@gmc.edu.cn (Y.W.); 2 School of Pharmacy, Guizhou Medical University, 4 Beijing Road, Guiyang 550004, China; 3 Engineering Research Center for the Development and Application of Ethnic Medicine and TCM, Ministry of Education, 4 Beijing Road, Guiyang 550004, China; gywam100@163.com (A.W.); yanyu626@126.com (Y.L.)

**Keywords:** gallic acid, pharmacokinetics, *Polygonum capitatum* extract, protocatechuic acid, pyelonephritis, quercitrin, UPLC-MS/MS

## Abstract

*Polygonum capitatum* Buch.-Ham. ex D. Don is traditionally used by Hmong for the treatment of urinary tract infections and pyelonephritis. Information regarding the pharmacokinetic behavior of the extract in the condition of pyelonephritis is lacking. In the present study, we aimed to compare the pharmacokinetic properties of gallic acid (GA), protocatechuic acid (PCA), and quercitrin (QR)—the main bioactive constituents in the herb—in normal and pyelonephritis rats. The plasma samples were collected at various time points after administration of a single dose of *Polygonum capitatum* extract. The plasma level of GA, PCA, and QR at the designed time points was determined by ultra-performance liquid chromatography-tandem mass spectrometry (UPLC-MS/MS) and drug concentration versus time plots were constructed to estimate the pharmacokinetic parameters. The AUC_(0-t)_, AUC_(0-∞)_, MRT_(0-t)_, and CL of GA, PCA, and QR in pyelonephritis rats was significantly different from those of the normal rats. The results indicated that the three constituents have higher rate of uptake and slower rate of elimination in the rats with pyelonephritis, suggesting altered rate and extent of drug metabolism.

## 1. Introduction

Urinary tract infection is one of the most common diseases, and is most frequently caused by *Escherichia coli*. The clinical manifestations of this infection range in severity from cystitis to acute or chronic pyelonephritis (PYN). If not treated appropriately, it can lead to permanent renal damage [1]. The disease might change the process of treatment, affecting the efficacy and adverse reactions of a drug. For instance, the half-life of the antihypertensive drug captopril in patients with renal function was approximately 12 times higher than that in a normal person [2]. The pharmacokinetic data of a drug are basically obtained from healthy subjects. If the changes in pharmacokinetics under disease conditions are not taken into account, serious adverse reactions might occur. Therefore, the study of pharmacokinetics under different disease conditions has attracted attention globally.

**Figure 1:**
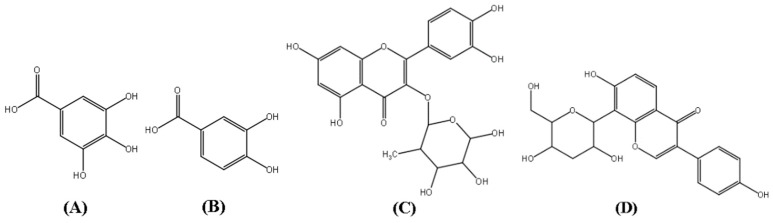
Structures of gallic acid (GA, **A**), protocatechuic acid (PCA, **B**), quercitrin (QR, **C**), and puerarin (**D**, internal standard).

*Polygonum capitatum* (Chinese name: *Tou-hua-liao*, Latin name: *Polygonum capitatum* Buch.-Ham. ex D. Don), belonging to the family Polygonaceae, is a well-known Miao herb. The plant has been widely used in China for the treatment of urinary tract infection, PYN, cystitis urinary calculi, and other disease [3,4]. Its preparation (as Re-Lin-Qing granules) is widely used in the clinics in China [5]. Modern pharmacological studies have indicated that the aqueous extract of *Polygonum capitatum* possesses antioxidant [6], antibacterial [7], and anti-inflammatory activities [8]. In the early stage of the research group, the chemical composition and pharmacological effects of the *Polygonum capitatum* were systematically studied, and some results were obtained. Chemical research showed that it mainly contains two major types of flavonoids and phenolic acids. The results of related pharmacodynamic experiments showed that these two components may be the main active constituents of the *Polygonum capitatum* [4,8,9,10] Among them, gallic acid (GA), protocatechuic acid (PCA), and quercitrin (QR) [11] (Figure 1) have been known to contribute to the major clinical therapeutic efficacies of *Polygonum capitatum* [9,12,13]. Phenolic compounds are well known secondary metabolites with broad pharmacological activity [14]. The antioxidant properties of these compounds have been studied and these oxidative properties lead to various degenerative diseases such as cardiovascular disease [15], inflammation [16,17] and cancer [18].

The results of previous studies showed that the endogenous creatinine clearance significantly decreased in the rats with PYN [19]. However, the pharmacokinetic properties of *Polygonum capitatum* extract in animals with PYN and the differences between normal animals and PYN model animals are seldom reported. The objective of this study was to investigate the possible pharmacokinetic differences in the constituents after the oral administration of *Polygonum capitatum* extract in normal rats and PYN model rats. It is believed that the study would provide valuable information for the effective use of *Polygonum capitatum* extract in clinic practice.

## 2. Results and Discussion

### 2.1. Evaluation of PYN Model

In the result of the evaluation of PYN model, the viscera indexes of rats all showed a certain increasing trend in the PYN model group, the positive detection rate of urine bacterial colony in the bladder was 100%. Blood routine tests found that both white blood cell count and neutrophil increase were consistent with the literature. Ccr and BUN can reflect the glomerular filtration function. When renal function damage such as glomerulonephritis and PYN, Ccr decreased, and Scr and BUN increased in concentration due to retention, which is an endogenous substance for evaluating renal function. NAG could reflect the damage of renal tubular function, and the activity of enzymes in urine was significantly increased when the renal tubules were damaged. TNF-α and IL-10 could reflect the production of inflammation, and TNF-α and IL-10 were significantly increased when inflammation occurs in the body. In this experiment, the levels of Scr and BUN in the infection model group were significantly increased, a large number of inflammatory factors were detected in the blood, and obvious inflammatory lesions appeared in the renal pathology, which was basically consistent with the literature reports, indicating that the experimental animal infection model was successfully prepared [20,21,22].

### 2.2. Method Validation

#### 2.2.1. Selectivity

**Figure 2:**
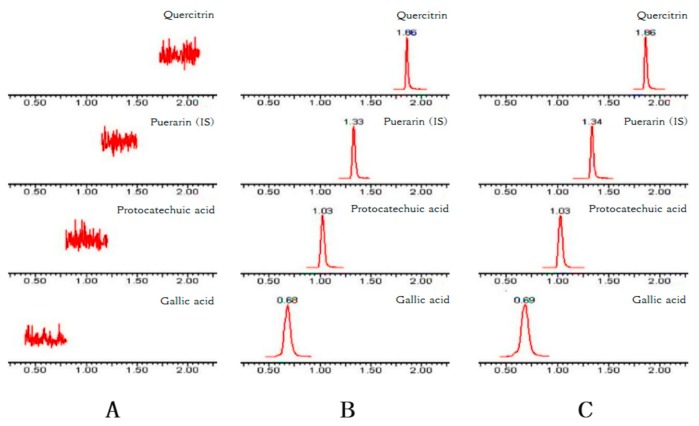
Representative chromatograms of GA, PCA, QR, and puerarin (IS) in (**A**) blank plasma; (**B**) blank plasma spiked with GA, PCA, QR, and IS; and (**C**) plasma at 30 min after the oral administration of 10 g/kg *Polygonum capitatum* extract.

Figure 2 shows the typical chromatograms of blank plasma (A), the plasma spiked with GA, PCA, QR, and the internal standard (IS) puerarin (B), and the plasma sample obtained from a rat 30 min after the intragastric administration of *Polygonum capitatum* extract (C). No interference from endogenous substances was observed at the retention time of the analytes and puerarin.

#### 2.2.2. Linearity and Lower Limit of Quantification

Each calibration curve was constructed with seven different concentrations by plotting the peak area ratio of puerarin versus the concentration of puerarin using linear regression. The typical calibration curves and linearity ranges of GA, PCA, and QR are listed in Table 1. The coefficients of correlation (*R*) of all the constituents were >0.9994. The lower limit of quantification (LLOQ) of the three analytes in the plasma is presented in Table 1.

#### 2.2.3. Precision and Accuracy

The intra- and inter-day precision and accuracy data of the three analytes in the quality control (QC) samples are shown in Table 2. The precision of the tested samples were within ± 15% (20% for LLOQ), meeting the acceptable criterion. The intra- and inter-day RSDs were all less than 9.88% and 9.12% (20% for LLOQ). These data demonstrate that the values are within the acceptable range and that the method used is accurate and precise.

#### 2.2.4. Extraction Recovery and Matrix Effect

The mean extraction recovery and matrix effect of the three constituents are presented in Table 2. The recovery of the three constituents was >87.18% at three levels, *viz.* low, medium, and high, indicating that the recovery of analytes was consistent and reproducible. The mean extraction recovery of puerarin was 95.02%. The matrix effects ranged from 93.42% to 98.17%, indicating no significant ion suppression or enhancement for the three analytes.

#### 2.2.5. Stability

The results presented in Table 3 reveal that the three constituents were stable under routine laboratory conditions.

### 2.3. Pharmacokinetic Studies

**Figure 3:**
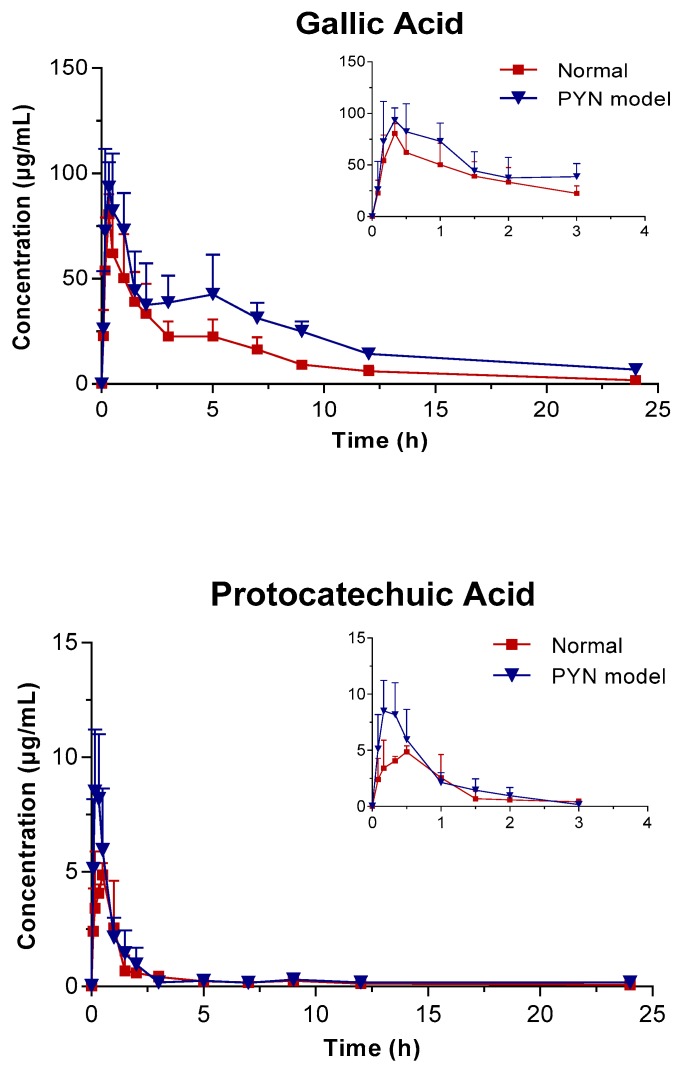

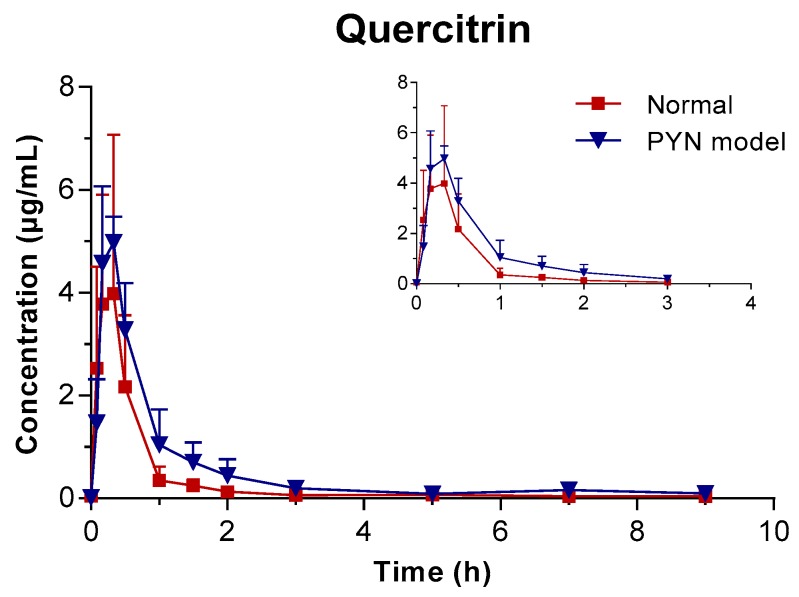
Mean plasma concentration vs. time profile of the three components following the oral administration of *Polygonum capitatum* extract in normal and PYN model rats (mean ± SD, *n* = 6).

The mean plasma concentration of GA, PCA, and QR versus the time profile following the oral administration of *Polygonum capitatum* extract is shown in Figure 3. The three constituents were absorbed rapidly in rats after intragastric administration. The main pharmacokinetic parameters were analyzed using the method of “3.11” and summarized in Table 4. The results were expressed as mean ± SD.

In comparison, the pharmacokinetics of GA, PCA, and QR changed significantly when *Polygonum capitatum* extract was administered to PYN model rats. There were statistically significant differences among the parameters, including the maximum plasma drug concentration (C_max_), area under the plasma drug concentration-time curve from 0 h to the terminal time point (AUC_(0-t)_), mean residence time (MRT_(0-t)_), and clearance (CL), between the normal and PYN model rats; both were administered equal amount of *Polygonum capitatum* extract containing GA (1.441%), PCA (0.043%), and QR (0.211%) [23]. Particularly, in the PYN model rats, the AUC_(0-t)_, AUC_(0-∞)_, MRT_(0-t)_, and C_max_ of GA, PCA, and QR increased significantly and the CL decreased significantly, compared with those of the normal animals. The results indicated that GA, PCA, and QR have a higher rate of uptake and slower rate of elimination in the animals with PYN.

The multi-component pharmacokinetics of traditional Chinese medicine are important aspects that are clarified by the theory and application of traditional Chinese medicine. However, due to the complexity and diversity of traditional Chinese medicine ingredients, as well as the complexity and uncertainty of the interaction between traditional Chinese medicine ingredients and biological systems, the research on multi-component pharmacokinetics of traditional Chinese medicine has been facing enormous challenges. And the drug is used to treat diseases, mainly in the disease states. Therefore, it is more important to study the pharmacokinetics under disease conditions than normal ones, and it is more relevant to the clinic [24].

In recent years, more and more studies have shown that the pharmacokinetic characteristics of traditional Chinese medicine will change under disease state. This is mainly due to this changes that may lead to changes in drug metabolism enzymes, cell membrane permeability, transport proteins, and microbial flora, thereby changing the absorption, distribution, metabolism, and excretion processes in vivo, and thus causing changes in the pharmacokinetic parameters of traditional Chinese medicine [25]. Just as the pharmacokinetics of the *Polygonum capitatum* extract in normal and PYN rats, the AUC, MRT and Cmax of three indicators increased in the PYN state while CL decreased. It was indicated that under PYN state, the three components could be absorbed into the blood more while could be eliminated slower in vivo, so that the residence time in vivo was prolonged and the drug effect could be better exerted.

The MRT of three indicator components such as GA in PYN rats was greater than that in normal rats suggesting that when the same drug was used in different individuals, the MRT might change due to differences in physiological and PYN state, and the rate of elimination in vivo changed. Therefore, clinical treatment should be based on the patient’s physiological and disease conditions to develop a reasonable dosing regimen, and special attention should be paid to drugs with narrow therapeutic concentrations. The half-life of the drug plays an important role in the selection and design of the drug dosage form and the determination of the clinical drug method [26,27].

In addition, PYN state can cause the decline of immune organ function and immunity, causing inflammation and with damage of kidney and tissue [19]. The kidney is the main excretory organ of the body. Kidney damage can lead to a decrease in the body’s excretion ability, which increases the retention time of the three components of PCA, GA and QR in vivo, and changes the pharmacokinetic process of the drug in vivo.

GA presented double peaks in the PYN model rats (Figure 3), which might be attributed to enterohepatic circulation. Gallic acid is transported from the liver to the small intestine via the bile duct, where they are largely reabsorbed through the cavity of the gastrointestinal tract back into the portal blood circulation. Therefore, the decline in the excretion capacity of the kidneys cannot be ruled out in time. Changes in the metabolic enzymes, transporters, and endogenous biological factors have been reported in patients with PYN model [28,29,30,31,32]. This might result in differences in the pharmacokinetic behavior between PYN and normal rats after the oral administration of *Polygonum capitatum* extract. However, this hypothesis is untested and needs to be validated by further studies. 

In general, the increase in the systemic exposure of the three active ingredients in the *Polygonum capitatum* extract in the PYN rats was improved, and the reason for this phenomenon was reduced renal function due to PYN [33]. In addition, after reviewing the literature, we believe that there are two potential mechanisms: (1) The stress of the body’s PYN state (kidney inflammation) decreases the endogenous creatinine clearance rate, the renal tubule reabsorption function and the glomerular filtration rate function, which ultimately reduces the excretion ability of drugs and increases the exposure in vivo. (2) Under the condition of PYN, it may cause changes in the internal environment such as gastrointestinal metabolism, intestinal blood flow and peristaltic function [34,35,36], which may prolong the residence time of the drug in the gastrointestinal tract and increase the absorption in the gastrointestinal tract. At the same time, adverse reactions may occur. However, this assumption needs further research to verify.

## 3. Materials and Methods 

### 3.1. Materials and Reagents

Reference substances GA (product no. 110831-201605), PCA (product no. 110809-201604), and QR (product no. 111538-201606), and the internal standard puerarin (product no. 110752-201615) were purchased from the China National Institutes for Food and Drug Control (Beijing, China). Mueller Hinton (MH) broth (product no. 20160213) was procured from Qingdao Hope Bio-Technology Co. Ltd. (Qingdao, China). *Escherichia coli* strain ATCC 25,922 was obtained from the Guangdong Microbial Culture Center (Guangzhou, China). High-performance liquid chromatography grade acetonitrile, formic acid and methanol were purchased from Merck & Co., Inc. (Germany). Distilled water was obtained from Guangzhou Watson’s Food & Beverage Co., Ltd. (Guangzhou, China). All other solvents used were of analytical grade and are commercially available.

In August 2016, the whole plant of *Polygonum capitatum* was collected from the Good Agricultural Practice (GAP) Base of *Polygonum capitatum* in Shi Bing (Guizhou, China). The specimen (accession No. PC 20160922) has been deposited in the Herbarium of Guizhou Medical University for reference.

### 3.2. Polygonum Capitatum Extract Preparation

A total of 5 kg of dried *Polygonum capitatum* whole plants was boiled in 50 L of water for 2 h and then filtered. The residual plants were added to 40 L of water and boiled for another 2 h. The two extracts were subsequently combined, dried, and ground and the resulting powder was used for the subsequent analysis. The extraction yield was 17.6% (g/g) [23], and the contents of GA, PCA, and QR were determined by UPLC-MS/MS). 

### 3.3. Animals and Treatments

Female Sprague-Dawley rats (220 ± 20 g) with no specific pathogen were provided by the Experimental Animal Center of Guizhou Medical University (Certificate no. SYXK 2018-0001). Prior to the experiments, the rats were acclimatized for a week in an animal room under air conditioning (22 ± 2 °C) and an auto-controlled photoperiod of 12:12 h light/dark cycle. The rats were fed with food and water *ad libitum*. The experimental procedures were in accordance with the Guidelines of the China Laboratory Animal Care and Use Committee. Twelve rats with similar average body weight were divided into two groups (six animals per group); a normal group (without any treatment) and PYN model group (bacterial inoculum was introduced into the bladder of the rats at 24 h after intraperitoneal sterile saline injection). After 3 d, the rats in both normal and PYN model groups received *Polygonum capitatum* extract at a dose of 10 g/kg. Approximately 250 μL of blood was drawn from the jugular vein in a plastic centrifuge tube coated with heparin before the administration and at 0.08, 0.17, 0.33, 0.5, 1, 1.5, 2, 3, 5, 7, 9, 12, and 24 after administration, while heparin sodium solution (50 IU/mL) was added into the tube. The blood samples were centrifuged at 4500 rpm for 10 min, following which 100 μL of upper plasma were separated and stored in a refrigerator at −20 °C until analysis.

### 3.4. Establishment and Evaluation of PYN Model 

The PYN rats were induced by ATCC 25,922 of *Escherichia coli* (*E. coli*) strain. *E. coli* cells were cultured in MH broth at 37 °C overnight until harvested. According to the method of Bennett et al. [37,38,39], the cells were diluted to a final concentration of approximately 10^10^ organisms/mL in 1× phosphate-buffered saline, and subsequently determined by the optical density at 600 nm.

The solution with 10^10^ bacteria/mL was diluted four times using sterile saline solution. The rats were anesthetized with chloral hydrate [40], and the bacterial suspensions were administered to the rats on the day PYN model was prepared. A sterile 22-gauge angiocatheter sheath was inserted into the urethra, and the bladder was emptied while 0.4 mL of solution was instilled: bacterial solution (1 × 10^9^ colony forming units) for PYN group and sterile saline solution for normal. The angiocatheter was then removed and the urethra was occluded with collodium. The collodium was removed with acetone after 4 h of bacterial inoculation [41,42,43].

The changes of renal weight coefficient, bladder coefficient, blood routine, hematuria creatinine (Cr), urinary bacterial culture, urea nitrogen (BUN), urinary β-N-acetylglucosaminidase (NAG), pathological sections, TNF-α, and IL-10 were compared between the two groups. The stability and reliability of the rat PYN model were evaluated to provide reference for the pharmacokinetic study of the *Polygonum capitatum* extract in normal and PYN rats [44,45].

### 3.5. Plasma Sample Preparation

To 100 μL of rat plasma sample, 20 μL of IS solution (5.50 μg/mL) and 50 μL of methanol were added, followed by 50 μL of 1% formic acid. The samples were then vortexed for 1 min. The mixture was extracted with 400 μL of methanol and vortexed for 3 min. After centrifugation at 12000 rpm for 10 min, the supernatant was transferred into another clean eppendorf tube quantitatively and evaporated to dryness at 37 °C with nitrogen. The residue was reconstituted in 150 μL initial mobile phase, and 5 μL the supernatant was injected into the UPLC–MS/MS system for analysis.

### 3.6. Stock and Working Solutions Preparation

A mixed stock solution contained with 1.018 mg/mL GA, 0.831 mg/mL PCA, and 0.426 mg/mL QR was prepared using methanol and successively diluted to the following concentrations for calibration curves: 0.2793–203.60 μg/mL, 0.0228–16.62 μg/mL, and 0.0195–14.20 μg/mL. All the solutions were stored at 4 °C before analysis.

### 3.7. Standard Solutions and Quality Control Samples Preparation

To yield calibration concentrations of 0.28, 0.84, 2.51, 7.54, 22.62, 67.87, and 203.60 μg/mL for GA; 0.02, 0.07, 0.21, 0.62, 1.85, 5.54, and 16.62 μg/mL for PCA; 0.02, 0.06, 0.18, 0.53, 1.58, 4.73, and 14.20 μg/mL for QR; and 5.50 μg/mL for IS, the standard solution was prepared by spiking 50 μL of appropriate mixed working solutions into 100 μL of blank plasma

The procedures of preparation of quality control (QC) samples at three different concentration levels were also similar to standard solutions. The QC samples were prepared at concentrations of 1.02, 20.36, and 203.60 μg/mL for GA; 0.08, 1.66, and 16.62 μg/mL for PCA; and 0.07, 1.42, and 14.20 μg/mL for QR. All the standard solutions were stored at 4 °C refrigerator and brought to room temperature before use. The QC samples were stored at −20 °C refrigerator.

### 3.8. Instruments and Analytical Conditions

The Acquity UPLC (TM) system tandem MS was used for separation and detection. The column used for chromatographic separation was a Waters Acquity BEH C18 column (2.1 × 50 mm^2^, i.d. 1.7 μm; Waters, Wexford, Ireland). The mobile phase was consisted of acetonitrile contained with 0.1% formic acid (A) as well as water contained with 0.1% formic acid (B). The elution gradient was as following sections: 5% A for 0 min, 30% A for 1.5 min, 90% A for 3.0 min, 5% A for 4.0 min, and 5% A for 5.0 min. The flow rate was 0.35 mL/min under 45 °C of the column temperature. The injection volume was 5 μL.

The Waters TQD Quantum triple-quadrupole mass spectrometer equipped with electrospray ionization (ESI) source was used for mass analysis and detection. The mass spectrometer was operated in either the positive or negative mode and multiple reaction monitoring (MRM) mode was selected to quantify the reference substrate (Table 5). Data acquisition and processing was performed using Micromass Masslynx version 4.1 and the reference substrate was quantified according to a validated UPLC-MS/MS method. The main operating parameters were set as follows: desolvation temperature, 350 °C; nebulizer gas (N_2_), 650 L/h; source heater, 120 °C; and the scan time for each analyte, 0.1s.

### 3.9. Method Validation

This method has been validated in terms of specificity, LLOQ, linearity, accuracy and precision, recovery, matrix effect, and stability according to the US-FDA Guidelines [46,47]. The specificity was assessed by comparing chromatograms of three different plasma samples: blank plasma from six different rats (A), blank plasma spiked with GA, PCA, QR, and IS(B), and plasma samples obtained after the oral administration of *Polygonum capitatum* extract (C). The LLOQ was determined according to the lowest concentration on the calibration curve. The acceptable accuracy was within ± 20% while the precision was below 20%.

The calibration curves were prepared by analyzing a mixture of three calibration standards. The concentration ranges of GA, PCA, and QR were 0.2793–203.60 μg/mL, 0.0228–16.62 μg/mL, and 0.0195–14.20 μg/mL respectively. Furthermore, these curves were fitted by a least-square linear regression of the ratio of the three analytes to IS peak area versus the normalized standard concentration with a weighed factor (1/*X^2^*). To determine accuracy and precision, QC samples were analyzed at three concentration levels (*viz.*, low, medium, and high) in five replicates during intra- (the same day) and inter-day (three consecutive days). The precision was defined as the relative standard deviation (RSD, %).

The extraction recoveries of the three analytes and IS were determined at three QC levels and at one concentration level, respectively. The calculation was performed by comparing the analyte standard peak areas obtained from the extracted samples with post-extracted samples spiked with the analytes. The matrix effect was evaluated by comparing the peak areas of the analytes in post-extracted spiked samples with those of the analytes dissolved in methanol at the same concentration. Analyte stability was assessed by analyzing the three replicate samples of QC samples at three levels under different storage conditions: 8 h at 24 °C, three freeze-thaw cycles (from −20 °C to 20 °C), and short-term stability (−20 °C for 24 h).

### 3.10. Pharmacokinetic Study

The validated method was applied to the pharmacokinetic study of the three active ingredients in the normal and PYN model rats after the intragastric administration of *Polygonum capitatum* extract. The two group of rats were housed individually under normal conditions. After fasting for 12 h, the rats in the normal and PYN model groups were administered *Polygonum capitatum* extract (containing GA, PCA, and QR at concentrations of approximately 2.65, 0.08, and 0.29 g/kg, respectively) at a dose of 10 g/kg by intragastric administration. Blood samples (0.25 mL) were collected from the jugular vein of each rat into heparinized tubes at 0, 0.083, 0.17, 0.33, 0.5, 1, 1.5, 2, 3, 5, 7, 9, 12, and 24 h after intragastric administration. The blood sample was centrifuged at 3000 rpm for 10 min at once, and the supernatant plasma was collected and stored at −80 °C until further treatment.

### 3.11. Pharmacokinetic Data Analyses

To determine the pharmacokinetic parameters of the three active ingredients, the concentration-time data were analyzed using the DAS 2.0 pharmacokinetic program with the non-compartmental method (Chinese Pharmacological Society, China). The comparisons of pharmacokinetic data between the two groups were performed using the SPSS (IBM SPSS Statistics 22 Developer, IBM Corp., NY, USA) software with analysis of variance. The results with a *p* value < 0.05 were considered statistically significant for all the tests. All the results are expressed as arithmetic mean ± standard deviation (SD).

## 4. Conclusions

We developed a selective, sensitive, and reliable UPLC-MS/MS method for the simultaneous determination of three constituents of *Polygonum capitatum* extract in normal and PYN model rats. The pharmacokinetic results indicated that there were significant differences in the pharmacokinetic parameters for the three analytes among the two groups. Overall, when compared with those in the normal group, the absorption of the three components in *Polygonum capitatum* extract increased and their excretion decreased. These results will be useful for future studies on *Polygonum capitatum* extract and for applications in clinical therapy.

## Figures and Tables

**Table 1:** Regression equation, linear range, and lower limit of quantification (LLOQ) of the three constituents in rat plasma.

Constituent	*Y* = A*X* + B	*R*	Linear Range (µg/mL)	LLOQ (µg/mL)
Gallic acid	*Y* = 0.1471*X* + 0.4686	0.9994	0.2793–203.6	0.2793
Protocatechuic acid	*Y* = 0.4496*X* + 0.0458	0.9998	0.0228–16.62	0.0228
Quercitrin	*Y* = 0.4218*X* - 0.0192	0.9995	0.0195–14.20	0.0195

**Table 2:** Summary of accuracy, precision, extraction recovery, and matrix effect data of GA, PCA, and QR in rat plasma (*n* = 5).

Analyte	Concentration (µg/mL)	Intra-Day (RSD, %)	Inter-Day (RSD, %)	Accuracy (%, mean ± SD)	Recovery (%, mean ± SD)	Matrix Effect (%, mean ± SD)
Gallic acid	1.02	9.88	8.91	96.11 ± 9.50	90.90 ± 5.88	94.22 ± 4.34
	20.36	8.40	9.12	100.43 ± 8.44	92.17 ± 4.89	93.66 ± 3.21
	203.6	5.89	4.63	100.00 ± 5.89	93.33 ± 7.29	94.79 ± 3.19
Protocatechuic acid	0.08	6.56	6.36	102.49 ± 6.72	89.35 ± 4.06	93.42 ± 2.80
	1.66	7.15	8.76	100.00 ± 7.15	87.18 ± 2.07	95.32 ± 9.58
	16.62	3.78	3.62	106.23 ± 4.01	88.65 ± 3.92	93.54 ± 2.36
Quercitrin	0.07	8.23	9.06	100.93 ± 8.31	92.76 ± 5.87	98.17 ± 6.73
	1.42	4.66	4.92	101.40 ± 3.18	89.42 ± 4.16	94.37 ± 4.17
	14.2	5.26	4.23	100.00 ± 5.26	88.53 ± 3.84	96.25 ± 8.30

**Table 3:** Stability of the three components in rat plasma under various storage conditions (mean ± SD, *n* = 5).

Component	Concentration Spiked (μg/mL)	8 h at 24 °C		Three Freeze-Thaw Cycles		Short-Term Stability (−20 °C, 24 h)	
		Precision (RSD, %)	Accuracy (%)	Precision (RSD, %)	Accuracy (%)	Precision (RSD, %)	Accuracy (%)
Gallic acid	1.02	12.24	94.51 ± 11.57	12.35	98.43 ± 12.16	13.86	97.65 ± 13.53
	20.36	10.28	97.99 ± 10.07	3.78	105.16 ± 3.98	4.58	102.95 ± 4.72
	203.60	7.34	97.96 ± 7.19	7.82	100.56 ± 7.86	1.98	103.95 ± 2.05
Protocatechuic acid	0.08	8.43	103.75 ± 8.75	7.41	101.25 ± 7.50	5.06	98.75 ± 5.00
	1.66	4.09	103.01 ± 4.22	1.79	101.20 ± 1.81	8.02	97.59 ± 7.83
	16.62	4.97	106.50 ± 5.29	3.87	107.34 ± 4.15	3.74	107.88 ± 4.03
Quercitrin	0.07	5.71	100.00 ± 5.71	8.45	101.43 ± 8.57	10.00	100.00 ± 10.00
	1.42	2.76	102.11 ± 2.82	2.78	101.41 ± 2.82	3.50	100.70 ± 3.52
	14.20	7.36	100.42 ± 7.39	4.39	102.68 ± 4.51	0.78	99.65 ± 0.77

**Table 4:** Pharmacokinetic parameters of GA, PCA, and QR in rat plasma following the oral administration of *Polygonum capitatum* extract at a dose of 10 g/kg (mean ± SD, *n* = 6).

Parameter		Gallic Acid		Protocatechuic Acid		Quercitrin	
		Normal Group	PYN Model Group	Normal Group	PYN Model Group	Normal Group	PYN Model Group
AUC_(0-t)_	mg·h/L	303.15 ± 81.41	559.57 ± 93.64 **	8.43 ± 2.03	12.09 ± 3.82	2.76 ± 1.33	4.72 ± 0.86 *
AUC_(0-∞)_	mg·h/L	311.76 ± 84.83	614.19 ± 74.95 **	9.30 ± 1.84	14.81 ± 5.69 *	2.94 ± 1.36	5.05 ± 0.84 **
MRT_(0-t)_	h	5.54 ± 0.52	7.18 ± 0.41 **	4.64 ± 0.98	5.06 ± 1.15	1.14 ± 0.20	1.58 ± 0.32 *
MRT_(0-∞)_	h	6.22 ± 0.84	10.48 ± 2.77 **	7.97 ± 4.10	11.25 ± 5.29	2.13 ± 0.84	2.45 ± 1.10
T_max_	h	0.33 ± 0.12	0.33 ± 0.17	0.53 ± 0.27	0.25 ± 0.14 *	0.22 ± 0.09	0.25 ± 0.09
CL	L/h/kg	9.01 ± 2.37	4.37 ± 0.59 **	8.92 ± 1.96	6.06 ± 2.16 *	118.43 ± 56.50	58.78 ± 9.42 *
C_max_	mg/L	81.12 ± 9.73	104.91 ± 9.67 **	5.61 ± 1.20	9.47 ± 3.14 *	5.07 ± 1.56	5.28 ± 0.81

Note: * *p* < 0.05, ** *p* < 0.01 compared with the PYN model group.

**Table 5:** List of selected MRM parameters, cone voltage (CV), collision energy (CE), and retention time of each analyte and internal standard (IS).

Analyte	Polarity	Q1 Mass (Da)	Q3 Mass (Da)	CV (V)	CE (eV)
Gallic acid	ESI-	169.00	125.00	35	15
Protocatechuic acid	ESI-	152.90	109.00	35	15
Quercitrin	ESI+	449.20	303.10	20	10
Puerarin	ESI+	417.15	267.10	40	30

## Abbreviations

AUC, area under the plasma drug concentration-time curve; C_max_, maximum plasma drug concentration; CL, clearance; ESI, electrospray ionization; GA, gallic acid; IS, internal standard; LLOQ, lower limit of quantification; MRT, mean residence time; PCA, protocatechuic acid; PYN, pyelonephritis; QR, quercitrin; QC, quality control; RSD, relative standard deviation; SD, standard deviation; T_max_, Peak time; TMC, traditional Chinese medicine; UPLC-MS/MS, ultra-performance liquid chromatography tandem mass spectrometry.
